# Process and effect evaluation of the app-based parenting program *Samen Happie!* on infant zBMI: A randomized controlled trial

**DOI:** 10.3389/fpubh.2022.1012431

**Published:** 2022-12-23

**Authors:** Levie T. Karssen, Junilla K. Larsen, William J. Burk, Stef P. J. Kremers, Roel C. J. Hermans, Emilie L. M. Ruiter, Jacqueline M. Vink, Carolina de Weerth

**Affiliations:** ^1^Behavioural Science Institute, Radboud University, Nijmegen, Netherlands; ^2^NUTRIM School of Nutrition and Translational Research in Metabolism, Maastricht University Medical Center, Maastricht, Netherlands; ^3^Academic Collaborative Centre AMPHI, Primary and Community Care, Radboud University Medical Centre, Nijmegen, Netherlands; ^4^Donders Institute for Brain, Cognition and Behaviour, Radboud University Medical Center, Nijmegen, Netherlands

**Keywords:** childhood obesity, preventive intervention, parenting practices, energy balance-related behavior, socioeconomic position (SEP), mHealth, behavior change

## Abstract

**Background:**

Although energy balance-related parenting practices are regarded critical components in the prevention of childhood obesity, most programs targeting parenting practices with respect to a wide range of energy balance-related behaviors were not aimed at high-risk families with a lower socioeconomic position (SEP).

**Objective:**

The *Samen Happie!* app-based program aimed to stimulate healthy child weight development especially among families with a lower SEP, by encouraging healthy energy balance-related parenting practices.

**Methods:**

A two-armed randomized controlled trial examined the process and effectiveness of the *Samen Happie!* program on child zBMI outcomes at 6- and 12-months follow-up. In total, 357 Dutch parents with infants aged 5–15 months old at baseline participated. Parents in the app condition (*n* = 179) received access to the *Samen Happie!* app and were compared to a waitlist-control condition (*n* = 178). Changes in zBMI were examined through linear mixed-effects models based on intention-to-treat and exploratory per-protocol principles.

**Results:**

Process data showed low levels of sustained app use and moderate app acceptability. A general increase in child zBMI was observed in both conditions after 6 and 12 months. Intention-to-treat analyses using multiple imputations showed several statistically significant differences between conditions and high-risk subgroups. Specifically, at 6-months follow-up, zBMI increase was least pronounced in the app condition among children of parents with lower educational level. These findings were supported by exploratory per-protocol analyses including only frequent app users. In addition, per-protocol analyses showed benefits of app use at 6-months follow-up for children of parents with higher BMI. However, these effects were reversed at 12-months follow-up in both intention-to-treat and per-protocol analyses, where children of parents in the app condition in general increased the most in zBMI.

**Conclusions:**

This study suggests that the *Samen Happie!* program might prevent zBMI increases after 6 months among children of parents with lower educational level, and children of parents with higher BMI who more frequently use the app. However, the app did not prevent increases in zBMI after 12 months. Future research should investigate strategies to increase sustained app use and engagement in mHealth parenting programs for childhood obesity as well as options to combine app-based programs with additional support strategies aimed at high-risk families.

**Trial registration:**

Netherlands trial register (ID: NTR6938), https://trialsearch.who.int/Trial2.aspx?TrialID=NTR6938.

## 1. Introduction

Childhood overweight and obesity remain urgent medical and societal problems that disproportionally affect children coming from families with a lower socioeconomic position (SEP). On average, 8% of Dutch children around the age of 2 had overweight or obesity in 2018 (1), but the prevalence varies based on parental educational level [i.e., a common indicator of SEP showing consistent inverse associations with child adiposity; (2, 3)]. Compared to children of higher-educated parents, Dutch children of parents with middle and lower educational level have 1.96- and 2.76-times higher risks of developing overweight and obesity in childhood, respectively (4). This SEP-gradient in adiposity, as well as the various obesity-related physical and psychosocial health consequences (5), the difficulty to treat the condition (6), the link between rapid weight gain in infancy and childhood overweight (7, 8), and the likelihood of obesity tracking into childhood and adulthood (9, 10) all emphasize the need for early obesity prevention tailored at families of parents with lower educational levels. As such, it is imperative that healthy energy balance-related behaviors [EBRBs: i.e., dietary intake, sleep, and physical (in)activity] underlying weight changes are established as soon as possible, during the first years of life (11). In these early years, children's EBRBs and subsequent weight status are predominantly managed and supported by their parents. This makes the stimulation of healthy energy balance-related parenting practices [i.e., specific, discrete, and observable acts of parenting related to child EBRB; (12)] a key component in early preventive interventions for childhood obesity (13). The goal of the present study is to test an innovative app-based preventive program for early childhood obesity addressing healthy energy balance-related parenting practices.

Of note, previous prevention programs have already shown potential positive effects on healthy weight outcomes by promoting parenting practices with respect to child dietary intake [e.g., responsive feeding (14–16), structure and rule setting (14)], and sleep [e.g., bedtime routines; (17)]. Moreover, reviews on parenting practices related to child physical activity suggest the importance of parental role modeling (18, 19). However, most early childhood prevention programs (i.e., < 5 years) targeting energy balance-related parenting have been limited in one of two ways. They either targeted some (but not all) EBRBs [i.e., most focused on dietary intake and/or physical activity; (16)] or they were universal (i.e., population-based) in nature, thereby not specifically directed at families that need it the most, including those with a lower SEP (16, 20, 21). As patterns of energy balance-related behaviors tend to cluster and unhealthier clusters are more frequently observed in families with a lower SEP [e.g., (22, 23)], targeting multiple EBRBs in families with lower SEP seems imperative. Our preventive parenting program aimed to overcome these limitations of previous studies by preventing childhood obesity through the stimulation of healthy parenting practices with respect to three important child EBRBs [i.e., child dietary intake, sleep, and physical (in)activity], while simultaneously applying selective prevention to a subgroup of parents with a lower SEP.

Our preventive parenting program was delivered *via* an app to facilitate the reach of deprived populations (24) and included relevant and engaging content and techniques through continuous co-creation with the target group. As such, our app-based program may specifically benefit families with a lower SEP that need the most help in terms of improving parenting practices and the home food environment (25–28). Moreover, two other risk groups that might also benefit more from the program are children of parents who have overweight and those of parents who experience mental health problems. To date, reviews have shown that children of parents with overweight or obesity have increased chances of developing overweight in childhood (29, 30). Although the exact mechanisms underlying this association are complex, it is presumed that a generally unhealthier home environment and more obesity-promoting parenting practices [e.g., parents with overweight apply less modeling of healthy food intake; (31)] can exacerbate the child's genetic predisposition for adiposity [e.g., (30, 32)]. Moreover, parental mental health problems can affect child development through various mechanisms [e.g., epigenetic processes during pregnancy (33), changes in breast milk (34)], including unhealthy parenting mechanisms in which mental problems impact parents' own EBRBs and reduce parents' responsiveness to their children's needs (35). Specifically, mothers who experience depressive symptoms more often apply unhealthy parenting practices such as parental modeling of unhealthy food intake, using food as a reward, and providing less structured sleep and activity-time (35–39). Notably, as our program targets particularly those parenting practices that are often less optimal among parents with obesity and depressive symptoms, children of these parents might also benefit more from the program. In addition, the app also targeted parents' own wellbeing by offering strategies to reduce stress and enjoy parenting (e.g., mindful parenting), and children of parents with depressive symptoms might experience benefits because of this as well. As the program aims to facilitate both child health and parental wellbeing and focuses for a large part on child dietary intake, we gave the program a Dutch title with a double meaning: *Samen Happie!*. Literal translations are “Happy Together” or “Eating Together,” but neither of these titles reflect the play on words in the Dutch language. We will therefore use the Dutch title throughout the paper.

### 1.1. The present study

The present study examined the effectiveness of the *Samen Happie!* app-based program in terms of reach, use, acceptability, and child zBMI among Dutch parents and infants (aged 5–15 months at the start of the program). We hypothesized that children of parents who used the *Samen Happie!* app would have a lower zBMI at 6 and 12 months after the start of the program than children of parents who did not use the app. Furthermore, we expected that these effects on child zBMI after 6 and 12 months would be particularly strong for children of parents who had a lower educational level, higher BMI, and more depressive symptoms.

## 2. Materials and methods

This randomized controlled intervention study employed a between-participants design with two parallel conditions: an app-based intervention condition and a waitlist-control condition. This study is part of a larger preventive intervention program being evaluated in two separate trials (i.e., Trial 1 included parents of infants and Trial 2 included parents of toddlers), both of which have been published together as study protocol (40). The present study focused on the process and effectiveness of the program in Trial 1. Key elements of the protocol pertaining to this trial are described below. The methods, materials, and analyses for this study were pre-registered (https://osf.io/hfvda).

### 2.1. Study participants and procedures

Trial 1 of the *Samen Happie!* program was conducted in the Netherlands between January 2018 and November 2019. Parents were eligible for participation in this trial if their child was between 5 and 15 months old at baseline and did not suffer from chronic disease or disability that severely affected normal development (e.g., chromosomal disorders, diabetes, cystic fibrosis), as indicated by parents in a web-based screening. We asked the primary caregiver of a child to participate in the trial. We further strived to include a minimum of 300 parents, of whom at least 50% had a middle or lower educational level [see study protocol (40) for power calculations]. Participants were recruited both offline (e.g., through child day care centers and preventive child health clinics for young children) and online (e.g., through Facebook groups). A diversity of locations and websites were used to ensure recruitment took place among parents of all educational levels. Eligible parents who completed the screening were forwarded to a consent form and subsequent baseline questionnaire. Parents were enrolled in the trial when they completed the web-based screening, the consent form, and the baseline questionnaire. Parents in both conditions were told that the aim of the trial was to investigate ways to assist families in healthy parenting, and that half of the study participants would receive an app that could help with healthy parenting. Parents that were allocated to the waitlist-control condition knew that they would receive access to the app at the end of the trial. The procedures of the trial were approved by the Ethics Committee Social Sciences, Radboud University, the Netherlands (ECSS-2017-013).

**Figure 1:**
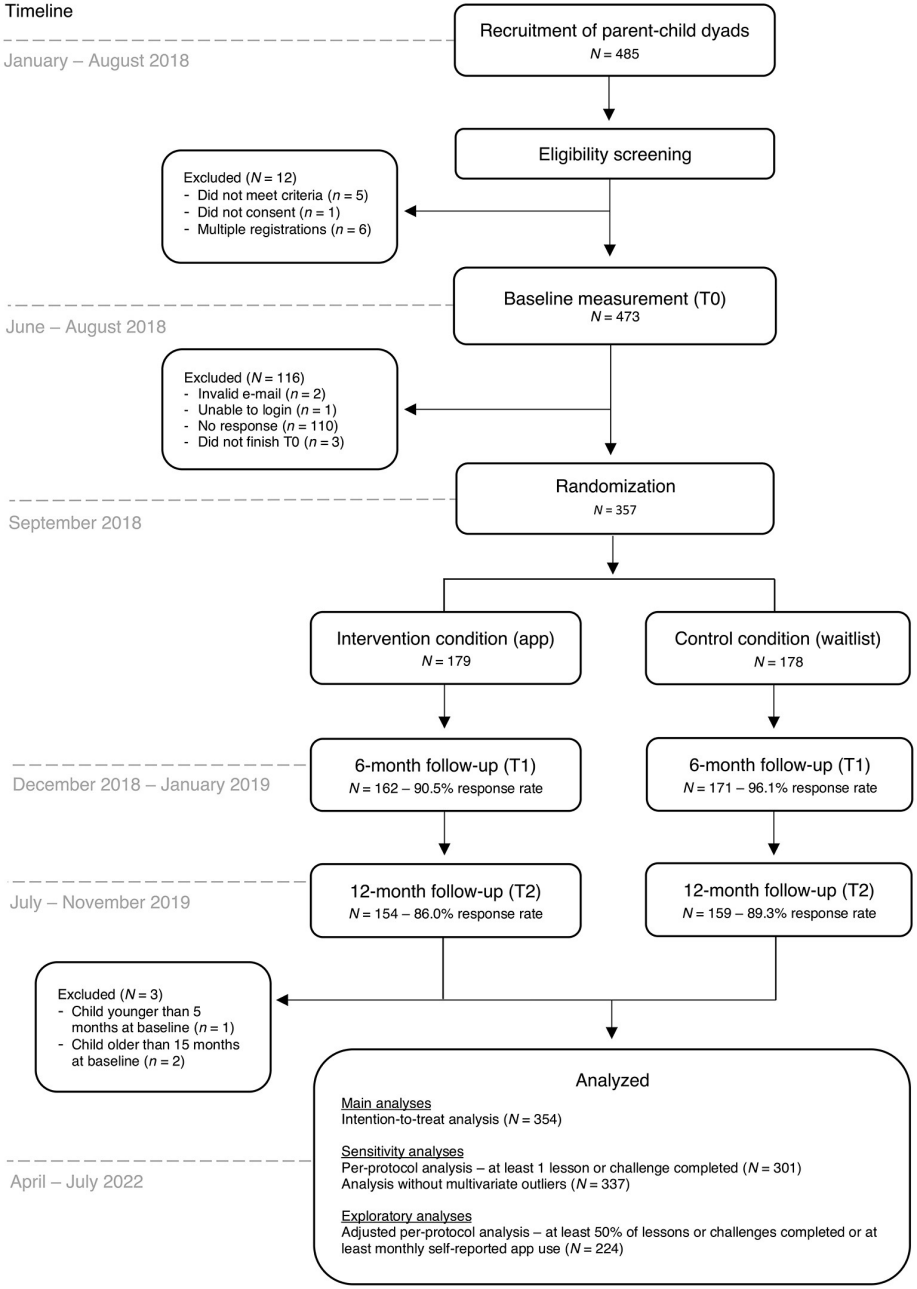
Flow chart of the *Samen Happie!* trial including information on its design, timing, and number of participants.

Randomization of the enrolled parents took place in September 2018. A simple randomization procedure (i.e., a computer-generated list of random numbers) to randomly allocate participants to the app or control condition (allocation ratio 1:1) was performed by an independent researcher using SPSS version 24. Among research with larger sample sizes (*N* > 200), this procedure can be trusted to produce equal samples in terms of numbers and covariates (41, 42). Parents who were allocated to the app condition received a personal invitation code for the *Samen Happie!* app and instructions on how to download and use the app. There were no instructions regarding the timing and frequency of the use of the app to stay as close as possible to app usage patterns in everyday life. A visual representation of the trial flow and its timing is presented in Figure 1.

### 2.2. The *Samen Happie!* program

The *Samen Happie!* program was developed using the Intervention Mapping Protocol (43), which included the integration of theory and empirical evidence as well as data from and continued co-creation with the target population. The program specifically addressed the needs of parents with a lower SEP through tailored program content and theory-based behavior change techniques, which were selected from behavior change taxonomies by Kok et al. (44) and Michie et al. (45). The program was delivered *via* a stand-alone, easy-to-use app consisting of five age-based modules: 7–12, 12–15, 15–18, 18–24, and 24–28 months. All parents in Trial 1 started in one of the first three modules at baseline. When the child reached the minimum age of the subsequent module, the new module became unlocked. Each age-based module provided parents with information (i.e., lessons) and exercises (i.e., challenges) about healthy parenting practices with respect to child EBRBs, as well as parental wellbeing and child temper (only lessons). Information in the lessons was presented in an engaging and easy-to-comprehend way, for instance through facts (“Did you know…?”), practical examples, tips, and quizzes, and was supported by icons and pictures. The challenges consisted of exercises that prompted parents to apply the information from the lessons in their day-to-day life. By employing techniques that tackle (unhealthy) automatic behaviors, parents were encouraged to implement (newly learned) parenting skills as habits. A more detailed description of the development, design, and content of the program can be found in the study protocol (40).

### 2.3. Data collection

Data were collected *via* web-based questionnaires at three timepoints: before the start of the program (T0), and after ~6 (T1) and 12 months (T2; see Figure 1 for the timing of the questionnaires). Non-responders were sent reminders *via* e-mail every 2 weeks during the measurement periods, with a maximum of 10 reminders at T2. Participants were compensated for their time and effort with a €10 gift card or a pack of diapers upon completing each questionnaire. Mean ages of the children at each questionnaire were 9.85 (*SD* = 2.24), 15.37 (*SD* = 2.54), and 22.87 (*SD* = 2.47) months at T0–T2, respectively.

#### 2.3.1. Assessment of child and parent characteristics

##### 2.3.1.1. Sociodemographic characteristics

Sociodemographic characteristics of the child that were collected at baseline (T0) included age, sex, and whether the child was first-born. Regarding breastfeeding, parents reported whether the child was ever breast-fed and the duration of breastfeeding (in months) at each measurement (T0-T2). For the parent, the following sociodemographic characteristics were collected at T0: age, relationship to child (i.e., biological father, biological mother, or other: foster, adoptive or other non-biological father/mother, partner of the biological father/mother, grandfather/grandmother, or guardian), educational level (i.e., primary school, preparatory vocational education, vocational education, pre-university, university), country of birth, parental relationship status, employment status, and financial difficulty (i.e., having difficulty paying bills over the past year). Parental educational level was dichotomized as lower (i.e., primary school, preparatory vocational education, vocational education) and higher educational level (i.e., pre-university and university).

##### 2.3.1.2. Parental depressive symptoms

Parental depressive symptoms were assessed using the Edinburg Postnatal Depression Scale [EPDS; (46)], which is a brief and highly acceptable self-report scale that can be reliably be used in non-postnatal women with older children (47). We used the Dutch translation validated by Pop et al. (48). The questionnaire consists of 10 Likert-scale items (response categories: 0–3) in which parents report how they felt in the past 7 days (e.g., “I have felt sad or miserable”). A total score for depressive symptoms at T0 was calculated by summing the responses on all EPDS items (possible range: 0–30), with higher scores indicating more depressive symptoms. The scale showed good internal consistency (Cronbach's alpha = 0.83).

#### 2.3.2. Assessment of app use

App use was assessed in two ways. First, through self-reports in the two follow-up questionnaires (T1 and T2), including questions about whether parents had downloaded the app (and if not, why), whether they still had the app installed on their phone (and if not, why), and how many times they had used the app. Second, objective characteristics of parents' app use were collected in an online database (e.g., the number and type of lessons and challenges started and/or completed).

#### 2.3.3. Assessment of app acceptability

At T1 and T2, we asked parents about their experiences with the app, including several indicators of functionality (e.g., ease of use), design, and content (e.g., usefulness) on a scale from 1 (bad experience) to 7 (good experience). These questions were adapted from the Mobile App Rating Scale (49). Parents also rated the app as a whole and indicated whether they would recommend the app to family or friends on a scale from 1 to 10, with higher scores indicating higher appreciation and a higher likelihood of recommendation. Finally, we asked open-ended questions about the ways in which parents thought the app could be improved.

#### 2.3.4. Assessment of parent and child anthropometry

Parents reported their height and weight at T0, from which we calculated parental BMI by dividing weight in kilograms by the squared height in meters. To assess child BMI, parents were asked to report the height and weight of the child as measured and reported by the youth health professional at their last visit to the preventive child health clinic (when available) at all three assessments. Parents were asked: “When was your child's height and weight measured at the preventive child health clinic for the last time?”, “What was the weight of your child in grams on that day?”, and “What was the length of your child in centimeters on that day?”. These questions were administered at T0–T2. Additionally, at the second follow-up (T2), parents were asked to send a picture or screenshot of the (digital) measurement overview provided by the preventive child health clinic (containing height and weight measurements from the moment of birth up until T2).

Standardized BMI scores at T0–T2 were calculated based on child height (in cm) and weight (in kilograms) using the anthro_zscores function of the anthro package (50) in R (51). Standardization according to child sex and age was based on WHO Growth Standards (52). Child height and weight were derived from parent reports at T0–T2. When entries were missing, we consulted data from the (digital) measurement overviews (if available). Correlations between parent reports and data from the measurement overviews were high (*r* = 0.95 at T0, *r* = 0.74 at T1, *r* = 0.89 at T2 for height and *r* = 0.96 at T0, *r* = 0.97 at T1, *r* = 0.79 at T2 for weight). Because parents reported the height and weight data based on scheduled visits to the preventive child health clinic, zBMI measurements did not always line up with the timing of the questionnaires. To calculate zBMI, we only used height and weight data (both parent-reported and from the measurement overview) that were measured no more than 3 months prior to the moment parents completed the questionnaire. Mean ages of the children at the zBMI measurements were 8.98 (*SD* = 2.47), 14.18 (*SD* = 2.30), and 22.22 (*SD* = 2.91) months at T0–T2, respectively.

### 2.4. Statistical analysis

Our hypotheses were tested with linear mixed-effects models to account for the nesting of repeated measures within participants. Four mixed effects models were performed in R (51) using the lmer function of the lmerTest package (53), which calls the lmer function of the lme4 package (54). Each model included a random intercept varying over participants (i.e., modeling per-participant random adjustments to the fixed intercept). To test the shorter-term and longer-term effects of the intervention, two time contrasts were created using simple contrasts: a shorter-term predictor comparing baseline vs. follow-up 1 (T0 coded as −1/3, T1 as +2/3, and T2 as −1/3) and a longer-term predictor comparing baseline vs. follow-up 2 (T0 coded as −1/3, T1 as −1/3, and T2 as +2/3). Contrast coding was also used for the categorical predictors condition (control condition coded as −1/2 and app condition as +1/2) and parental educational level (lower educational level coded as −1/2 and higher as +1/2). The model testing the first hypothesis (i.e., the overall intervention effect) included a fixed intercept, a fixed slope for the factor condition, a fixed slope for the factor shorter-term follow-up, a fixed slope for the factor longer-term follow-up, and two fixed slopes for the interactions between both time contrasts and condition. The model testing the moderating effect of parental educational level included all predictors of the first model plus the three-way interactions between both time contrasts, condition, and parental educational level, as well as the main effect of parental educational level and its lower-order interactions. The model testing the moderating effect of parental BMI included all predictors of the first model plus the three-way interactions between both time contrasts, condition, and parental BMI, as well as the main effect of parental BMI and its lower-order interactions. Lastly, the model testing the moderating effect of parental depressive symptoms included all predictors of the first model plus the three-way interactions between both time contrasts, condition, and parental depressive symptoms, as well as the main effect of parental depressive symptoms and its lower-order interactions. The continuous predictors parental BMI and parental depressive symptoms were centered.

Following the CONSORT guidelines for reporting RCTs (55), the analyses were performed according to the intention-to-treat (ITT) principle (i.e., including all randomized participants adhering to the inclusion criteria). To assess the robustness of the findings, two types of sensitivity analyses were performed for all four models: (1) analyses excluding multivariate outliers identified using Mahalanobis distance [where observations > 3 *SD*s from the mean were considered outliers; (56)], and (2) per-protocol (PP) analyses including only participants in the app condition who completed at least one lesson or challenge (vs. the control condition). Statistical significance of parameter estimates was determined based on *p*-values provided by lmerTest. Coefficients were considered statistically significant if *p* < 0.05. Confidence intervals were determined using lme4's confint function using bootstrapping with 1,000 simulations. Statistically significant interaction effects were interpreted by extracting and comparing estimated means using the emmeans function from the emmeans package (57).

During the analysis process, we observed that the outcome variable child zBMI contained a considerable amount of missingness (see Study Participants in the Results section for details). We therefore imputed missing data *via* multivariate imputations by chained equations using the mice function of the mice package (58). Twenty imputations were performed within the app and control condition separately and the results of the individual imputations were pooled using mice's pool function. Both the results of the analyses in the non-imputed and in the imputed data are presented in the paper.

#### 2.4.1. Deviations from pre-registration

We deviated from the pre-registration in two ways. First, *p*-values for the parameter estimates were derived *via* the lmerTest function instead of the mixed function from the afex package (59) for reasons of consistency across imputed and non-imputed analyses. This means that degrees of freedom were calculated *via* the Sattherwaite method, instead of the Kenward-Roger approach [which have shown to produce similar results; (60)]. Second, we unintentionally failed to specify the two-way interactions between each of the moderators and time and condition (e.g., parental educational level ^*^ time, parental educational level ^*^ condition) in the pre-registration, and included all possible lower-order interactions in the final analyses.

#### 2.4.2. Exploratory analyses

Based on observed differences in participants' program use (see App Use for details) we performed exploratory, more stringent PP analyses to explore intervention effects among participants with higher program use (vs. the control condition) in addition to our pre-registered (less stringent) per-protocol analyses. The initially planned PP analyses included participants in the app condition who completed at least 1 lesson or challenge during the program period (vs. participants in the control condition). On hindsight, this criterium for program use might have been too loosely specified. For the app to have an effect, parents should have been exposed to a substantial amount of app content and/or have engaged with the content frequently. Therefore, additional, more stringent PP analyses were explored including participants who (1) completed at least 50% of the available lessons at T2 (*n* = 19), or (2) completed at least 50% of the available challenges at T2 (*n* = 29), or (3) reported to use the app at least monthly at T1 (*n* = 27). Due to overlap in participants between the three criteria, a total of 47 participants with higher app use were included in the adjusted PP analyses and compared to the control condition (*n* = 177). These exploratory PP analyses were performed in both the non-imputed and imputed data.

## 3. Results

### 3.1. Study participants

A total of 485 parents were recruited; however, 128 parents were not enrolled in the trial for different reasons, for example because they did not complete the baseline questionnaire (see Figure 1). The final randomized sample included 357 primary caregivers (179 in the app condition and 178 in the control condition; see Figure 1). No parents were excluded based on their child's health condition, as the conditions that parents specified (e.g., cow's milk allergy and/or reflux, skin conditions such as eczema) did not severely impact the child's (weight) development. Three parents were excluded from the analyses because their child was younger than 5 months or older than 15 months at baseline, resulting in a final analytic sample of 354 participants (177 in the app condition and 177 in the control condition).

Retention in the study was high, with response rates of 94.1% (333/354) at T1 and 88.4% (313/354) at T2. A considerable amount of missingness was observed in the outcome variable child zBMI at T1 and T2 (42.6% (142/333) at T1 and 64.9% (203/313) at T2), which probably resulted from our request to report child length and height based on measures reported at the preventive child health clinic. These measurements are scheduled at specific ages (e.g., at 5, 9, 11, and 14 months) and become less frequent over time (i.e., after 18 months there are only yearly visits at 2, 3, and 4 years), therefore potentially resulting in missingness at specific ages. Independent samples *t*-test showed that children with missing data on zBMI at T2 were on average 22.20 months old (*SD* = 2.09), whereas those who did not have missing data on zBMI were significantly older [*M* = 23.55, *SD* = 2.63, *t*_(303)_ = 4.95, *p* < 0.001] and closer in age to the standard visit to the preventive child health clinic at 2 years. Participants with and without missing values for child zBMI at T1 and T2 did not differ based on child sex, age of the parent, BMI of the parent, parental depressive symptoms, parental educational level, parental employment status, and parental financial difficulty (*p*'s > 0.05).

### 3.2. Baseline characteristics

Descriptions of parent and child characteristics at baseline across the app and control conditions are provided in Table 1. Correlations between these variables at baseline are presented in Table 2. We found a positive but small association between child age and child zBMI (*r* = 0.13), indicating that being older was associated with higher zBMI scores. No other statistically significant associations between the outcome variable zBMI and parent or child characteristics at baseline were observed.

**Table 1:** Baseline parent and child characteristics by intervention condition.

**Characteristic**	**App condition (*n* = 177)**	**Control condition (*n* = 177)**
**Parent**		
Age in years, *M* (*SD*)	30.31 (4.13)	30.23 (3.93)
Relationship to child, *n* (%)		
Biological mother	171 (96.61)	171 (96.61)
Biological father	4 (2.26)	4 (2.26)
Other	2 (1.12)	2 (1.12)
In a romantic relationship, *n* (%)		
Yes, cohabiting with partner	167 (94.35)	165 (93.22)
Yes, not cohabiting with partner	3 (1.69)	3 (1.69)
No	7 (3.95)	9 (5.08)
Educational level^a^, *n* (%)		
Lower	86 (48.59)	88 (49.72)
Higher	91 (51.41)	89 (50.28)
Employment status, *n* (%)		
Employed	143 (80.79)	144 (81.36)
Not employed	34 (19.21)	33 (18.64)
Country of birth, *n* (%)		
Netherlands	171 (96.61)	170 (96.05)
Outside Netherlands	6 (3.39)	7 (3.95)
Difficulty paying bills, *n* (%)		
Yes	13 (7.34)	14 (7.91)
Somewhat	90 (50.85)	85 (48.02)
No	74 (41.81)	78 (44.07)
BMI, *M* (*SD*)	26.18 (5.59)	26.03 (5.19)
Underweight, *n* (%)	2 (1.16)	3 (1.74)
Normal weight, *n* (%)	92 (53.18)	85 (49.42)
Overweight, *n* (%)	42 (24.28)	49 (28.49)
Obese, *n* (%)	37 (21.39)	35 (20.35)
Depressive symptoms, *M* (*SD*)	5.81 (4.52)	5.32 (4.04)
**Child**		
Age in months, *M* (*SD*)	9.67 (2.26)	10.03 (2.21)
Sex, *n* (%)		
Boys	97 (54.80)	89 (50.28)
Girls	80 (45.20)	88 (49.72)
First-born child, *n* (%)		
Yes	108 (61.02)	102 (57.63)
No	69 (38.98)	75 (42.37)
Ever breastfed, *n* (%)		
Yes	133 (75.14)	125 (70.62)
No	44 (24.86)	52 (29.38)
Breastfeeding duration in months, *M* (*SD*)	3.69 (4.03)	3.81 (4.23)
zBMI, *M* (*SD*)	−0.09 (1.03)	−0.08 (1.10)

^*a*^Parental educational level was dichotomized as lower (i.e., primary school, preparatory vocational education, vocational education) and higher educational level (i.e., pre-university and university).

**Table 2:** Pearson correlations between parent and child characteristics at baseline.

	**1**	**2**	**3**	**4**	**5**	**6**	**7**	**8**	**9**	**10**	**11**	**12**	**13**
1. Condition													
**Parent**				
2. Age	0.01												
3. Relationship to child^a^	0.00	0.07											
4. Romantic relationship^b^	−0.03	−0.04	0.03										
5. Educational level^c^	0.01	0.24^***^	0.06	−0.06									
6. Employment status^d^	−0.01	0.01	0.09	−0.21^***^	0.29^***^								
7. Born in the Netherlands^e^	−0.02	0.03	0.05	−0.04	0.01	−0.06							
8. Financial difficulty^f^	−0.02	0.10	0.03	−0.11^*^	0.33^***^	0.22^***^	0.01						
9. BMI	0.01	−0.16^**^	−0.04	−0.04	−0.28^***^	−0.13^*^	−0.06	−0.17^***^					
10. Depressive symptoms	0.06	−0.07	−0.14^**^	0.01	−0.11^*^	−0.17^**^	−0.05	−0.20^***^	0.07				
**Child**				
11. Age	−0.08	0.11^*^	−0.02	−0.03	0.08	0.14^**^	0.11^*^	0.09	−0.18^***^	−0.02			
12. Sex^g^	−0.05	0.06	0.04	−0.04	0.02	0.07	−0.01	0.07	0.01	−0.08	−0.01		
13. First born^h^	−0.03	0.21^***^	−0.03	−0.04	−0.08	−0.11^*^	−0.01	−0.08	−0.01	0.03	0.05	−0.02	
14. zBMI	0.00	−0.08	0.05	0.09	−0.02	−0.06	0.02	0.04	0.00	−0.01	0.13^**^	0.09	−0.07

Significance levels: ^*^p < 0.05, ^**^p < 0.01, ^***^p < 0.001.

^*a*^1, biological mother; 2, other caregiver.

^*b*^1, in romantic relationship; 2, not in romantic relationship.

^*c*^1, lower educational level; 2, higher educational level.

^*d*^1, not employed; 2, employed.

^*e*^1, born in the Netherlands; 2, born outside the Netherlands.

^*f*^1, difficulty paying bills; 2, no difficulty paying bills.

^*g*^1, boy; 2, girl.

^*h*^1, first-born child; 2, not first-born child.

### 3.3. App use

#### 3.3.1. Self-reported app use

After randomization, all 177 parents in the app condition received an invitation to download and use the app. At T1, 77.7% (122/157) of parents in the app condition reported they downloaded the *Samen Happie!* app. These numbers are comparable at T2 (75.3%, 113/150). The most frequently mentioned reasons for not downloading the app at T1 were that parents forgot to download it (51.4%, 18/35) or that they did not receive the e-mail with download instructions we sent them (31.4%, 11/35). At T1, 86.1% (105/122) of parents who downloaded the app reported that they still had the app installed on their phone, which decreased to 70.0% (70/113) at T2. Most parents who had the app installed on their phone at T1 and T2 reported that they used it several times after installing, but not anymore at T1 (70.5%, 74/105) or at T2 (78.6%, 55/70). Reasons for deleting the app (reported at T2) were (multiple answers allowed): the app did not work properly (2.3%, 1/43), the app was not interesting (41.9%; 18/43), I needed to free up phone storage (20.9%, 9/43), and I got a new phone (39.5%; 17/43). A smaller number of parents still used the app a couple of times per month [22.9% (24/105) at T1, 15.7% (11/70) at T2] or per week [2.9% (3/105) at T1, 2.9% (2/70) at T2].

#### 3.3.2. App use from database

Results from the database that collected data on parents' app use showed that parents started a total of 1,575 lessons and 406 challenges, of which 1,498 lessons (95.1%) and 381 challenges (93.8%) were completed. The number of lessons and challenges completed per parent in the app condition varied between 0 and 51 (*M* = 10.46, *SD* = 13.56). The mean number of lessons and challenges completed did not differ for parents with higher and lower educational level, as indicated by independent samples *t*-tests (*p*'s > 0.05). Most lessons and challenges were completed in module 1 (51.9%, 975/1,879) and the number of completed lessons and challenges decreased with each subsequent module: 21.8% (410/1,879) in module 2, 15.4% (290/1,879) in module 3, 10.2% (192/1,879) in module 4, and 0.6% (12/1,879) in module 5. Based on child age at baseline, the maximum number of lessons and challenges that could be completed at T2 was either 51 (67.8%, 120/177) or 70 (32.2%, 57/177). At T2, 30.5% (54/177) of parents completed at least 25% of the available lessons and challenges, 13.0% (23/177) completed at least 50% of the available lessons and challenges, and only 4.0% (7/177) completed at least 75% of the available lessons and challenges. Most lessons and challenges were completed within the theme food (36.8%, 691/1,879), followed by drinks (21.9%, 412/1,879), sleep (14.0%, 263/1,879) and parent wellbeing (14.0%, 263/1,879), screens or physical activity (PA; 7.4%, 139/1,879), summary (5.1%, 96/1,879), and temper (0.8%, 15/1,879). Supplementary Table 1 shows the number of available lessons and challenges per module and per theme, the mean number of completed lessons and challenges per module and per theme, and the number of parents with access to each module at T0, T1, and T2.

### 3.4. App acceptability

Means and standard deviations for the self-reported rating (scales ranging from 1 to 7) of different aspects of the app at T1 and T2 are presented in Table 3. Parents gave statistically significant higher scores for design of the app at T1 than at T2. No other differences were found for the app acceptability items at T1 and T2. Although scores on ease of use were high for all parents, parents with lower educational level rated the app somewhat lower (*M* = 5.02, *SD* = 1.77) than parents with higher educational level (*M* = 5.84, *SD* = 5.84) regarding ease of use at T1 (but not at T2), *p* = 0.003. The other acceptability ratings at T1 and T2 did not differ for parents with lower and higher educational level (*p*'s > 0.05). Overall, parents rated the app on average with a 6.60 (on a scale from 1 to 10) at both T1 and T2 (T1: *SD* = 1.42, T2: *SD* = 1.50), indicating moderate levels of acceptability of the *Samen Happie!* app. The average response to the question whether parents would recommend the app to others (on a scale from 0 to 10) was 5.80 (*SD* = 2.30) at T1 and 5.64 (*SD* = 2.28) at T2. The scores for app rating and intention to recommend did not significantly differ between T1 and T2 and did not differ for parents with lower and higher educational level, both at T1 and T2 (*p*'s > 0.05).

**Table 3:** Parent ratings of different aspects of the app at T1 and T2.

	**T1 (*n* = 122)**	**T2 (*n* = 113)**	
**The app is…**	***M*** **(*****SD*****)**	***M*** **(*****SD*****)**	***p***
Easy to use	5.48 (1.55)	5.53 (1.53)	0.426
Informative	4.48 (1.64)	4.50 (1.55)	0.656
Fast	5.33 (1.33)	5.31 (1.39)	0.264
Engaging	4.20 (1.56)	4.27 (1.73)	0.501
Nicely designed	5.38 (1.19)	5.11 (1.31)	**0.016**
Fulfilling my expectations	3.94 (1.51)	4.19 (1.48)	0.397
Making me feel confident	4.75 (1.19)	4.59 (1.24)	0.253
Useful	4.07 (1.51)	3.98 (1.76)	0.367
Clear	5.15 (1.21)	5.05 (1.36)	0.053
Helpful in my parenting	3.57 (1.56)	3.67 (1.78)	0.904

Ratings are on a scale from 1 to 7. Differences in ratings between T1 and T2 were tested using paired samples t-tests. Statistically significant comparisons (*p* < 0.05) are bolded.

### 3.5. Intervention effects on child zBMI

Tables 4, 5 present the results of the ITT analyses examining intervention effects on child zBMI using the non-imputed and imputed data. Table 4 shows that we found statistically significant and consistent main effects of time (both the shorter- and longer-term contrast) in all four analyses performed in the non-imputed and imputed data, indicating an increase in child zBMI over time and across conditions. Moreover, in the imputed data the two-way interactions between condition and both time contrasts (i.e., baseline vs. FU1 and baseline vs. FU2) were statistically significant. Table 5 presents the means and standard errors for child zBMI across conditions (i.e., overall intervention effect) and demonstrates that the increase in zBMI was greater in the control condition in the shorter-term, but greater in the app condition in the longer-term. These interactions did not emerge as statistically significant when using non-imputed data. Moreover, we found several statistically significant three-way interactions for subgroups based on parental educational level, BMI, and depressive symptoms in the imputed, but not the non-imputed data (see Table 4). First, we found interaction effects between parental educational level, condition, and both time contrasts. The estimated means in Table 5 show that in the shorter-term, zBMI increased the most among children of parents with lower educational level in the control condition and least among children of parents with lower educational level in the app condition. However, in the longer-term, the zBMI increases seemed more apparent in the app condition compared to the control condition, with the highest increases observed among children of parents with higher educational level. Second, there was an interaction between parental BMI, condition, and the longer-term time contrast. Table 5 shows that the longer-term increases in zBMI were highest in the app condition, particularly among children of parents with higher BMI. Third and finally, an interaction was observed between parental depressive symptoms, condition, and the longer-term time contrast. In the longer-term, zBMI increased in the app condition more than in the control condition, but children of parents with higher depressive symptoms in the control condition showed a similar increase in zBMI (see Table 5).

**Table 4:** Linear mixed model ITT intervention effects on child zBMI for subgroups based on parental educational level, BMI, and depressive symptoms in the non-imputed and imputed data.

	**Non-imputed**				**Imputed**			
	***b***	***SE***	**95% CI**	***p***	***b***	***SE***	**95% CI**	***p***
**Overall intervention effect**							
Condition	0.01	0.11	−0.21 to 0.24	0.937	0.06	0.09	−0.11 to 0.23	0.491
Shorter-term (baseline vs. FU1)	0.36	0.06	0.22–0.48	**< 0.001**	0.40	0.01	0.38–0.42	**< 0.001**
Longer-term (baseline vs. FU2)	0.39	0.07	0.26–0.54	**< 0.001**	0.40	0.01	0.38–0.43	**< 0.001**
Condition * shorter-term	−0.14	0.12	−0.36 to 0.12	0.257	−0.15	0.02	−0.20 to −0.11	**< 0.001**
Condition * longer-term	0.17	0.14	−0.12 to 0.44	0.247	0.33	0.02	0.29–0.38	**< 0.001**
**Parental educational level**							
Condition	0.01	0.11	−0.19 to 0.22	0.903	0.06	0.09	−0.10 to 0.27	0.468
Shorter-term (baseline vs. FU1)	0.36	0.06	0.24–0.48	**< 0.001**	0.40	0.01	0.37–0.42	**< 0.001**
Longer-term (baseline vs. FU2)	0.40	0.07	0.27–0.53	**< 0.001**	0.40	0.01	0.38–0.43	**< 0.001**
Parental educational level	−0.05	0.11	−0.25 to 0.17	0.620	−0.08	0.08	−0.25 to 0.08	0.325
Condition * shorter-term	−0.15	0.13	−0.41 to 0.09	0.229	−0.15	0.02	−0.20 to −0.11	**< 0.001**
Condition * longer-term	0.15	0.14	−0.13 to 0.46	0.289	0.33	0.02	0.29–0.37	**< 0.001**
Condition * parental educational level	−0.36	0.21	−0.77 to 0.06	0.092	−0.25	0.17	−0.59 to 0.09	0.145
Shorter-term * parental educational level	−0.09	0.13	−0.34 to 0.17	0.473	−0.10	0.02	−0.15 to −0.06	**< 0.001**
Longer-term * parental educational level	0.10	0.14	−0.19 to 0.39	0.506	−0.00	0.02	−0.04 to 0.05	0.979
Condition * shorter-term * parental educational level	0.35	0.25	−0.14 to 0.86	0.166	0.37	0.05	0.28–0.46	**< 0.001**
Condition * longer-term * parental educational level	0.07	0.29	−0.58 to 0.63	0.806	0.23	0.05	0.14–0.32	**< 0.001**
**Parental BMI**							
Condition	0.01	0.11	−0.19 to 0.23	0.912	0.06	0.09	−0.11 to 0.23	0.502
Shorter-term (baseline vs. FU1)	0.36	0.06	0.24–0.47	**< 0.001**	0.40	0.01	0.38–0.42	**< 0.001**
Longer-term (baseline vs. FU2)	0.40	0.07	0.26–0.55	**< 0.001**	0.40	0.01	0.38–0.43	**< 0.001**
Parental BMI	0.01	0.01	−0.01 to 0.03	0.343	0.01	0.01	−0.01 to 0.02	0.313
Condition * shorter-term	−0.14	0.13	−0.41 to 0.11	0.254	−0.15	0.02	−0.19 to −0.11	**< 0.001**
Condition * longer-term	0.17	0.14	−0.12 to 0.41	0.234	0.33	0.02	0.29–0.37	**< 0.001**
Condition * parental BMI	0.02	0.02	−0.02 to 0.06	0.427	0.01	0.02	−0.02 to 0.04	0.477
Shorter-term * parental BMI	0.02	0.01	−0.01 to 0.04	0.189	0.02	0.00	0.01–0.02	**< 0.001**
Longer-term * parental BMI	0.01	0.01	−0.02 to 0.03	0.641	0.01	0.00	0.01–0.02	**< 0.001**
Condition * shorter-term * parental BMI	0.00	0.02	−0.04 to 0.06	0.934	−0.01	0.00	−0.02 to −0.003	0.058
Condition * longer-term * parental BMI	0.01	0.03	−0.04 to 0.07	0.619	0.01	0.00	0.001–0.02	**0.034**
**Parental depressive symptoms**							
Condition	0.00	0.11	−0.20 to 0.20	0.985	0.05	0.09	−0.12 to 0.22	0.544
Shorter-term (baseline vs. FU1)	0.36	0.06	0.24–0.48	**< 0.001**	0.40	0.01	0.38–0.42	**< 0.001**
Longer-term (baseline vs. FU2)	0.41	0.07	0.27–0.56	**< 0.001**	0.41	0.01	0.39–0.43	**< 0.001**
Parental depressive symptoms	0.01	0.01	−0.01 to 0.04	0.327	0.01	0.01	−0.01 to −0.03	0.151
Condition * shorter-term	−0.15	0.13	−0.38 to 0.09	0.243	−0.16	0.02	0.28–0.37	**< 0.001**
Condition * longer-term	0.15	0.14	−0.11 to 0.45	0.285	0.32	0.02	−0.02 to 0.06	**< 0.001**
Condition * parental depressive symptoms	0.02	0.03	−0.03 to 0.07	0.466	0.02	0.02	0.02–0.03	0.361
Shorter-term * parental depressive symptoms	0.02	0.02	−0.01 to 0.05	0.209	0.02	0.00	0.02–0.03	**< 0.001**
Longer-term * parental depressive symptoms	0.02	0.02	−0.01 to 0.06	0.233	0.02	0.00	0.02–0.03	**< 0.001**
Condition * shorter-term * parental depressive symptoms	0.01	0.03	−0.04 to 0.07	0.628	0.00	0.00	−0.01 to 0.01	0.814
Condition * longer-term * parental depressive symptoms	−0.03	0.03	−0.10–0.03	0.317	−0.04	0.00	−0.05–−0.03	**< 0.001**

b, unstandardized regression coefficient; SE, standard error; CI, confidence interval. Parameter values for b and SE < 0.01 are presented as 0.00. Statistically significant effects (*p* < 0.05) are bolded.

**Table 5:** Estimated means (ITT) for child zBMI at baseline, follow-up 1, and follow-up 2 for the app and control condition, specified by the levels (i.e., lower vs. higher) of parental educational level, BMI, and depressive symptoms in the non-imputed and imputed data.

	**Non-imputed**								**Imputed**							
	**App condition**				**Control condition**				**App condition**				**Control condition**			
**Overall intervention effect**																
	***M***		***SE***		***M***		***SE***		***M***		***SE***		***M***		***SE***	
Baseline	−0.08		0.08		−0.08		0.08		−0.08		0.06		−0.08		0.06	
Follow-up 1	0.21		0.10		0.35		0.09		0.24		0.06		0.39		0.06	
Follow-up 2	0.40		0.11		0.24		0.11		0.49		0.06		0.16		0.06	
**Pairwise comparisons**			Shorter-term (baseline vs. FU1) change in zBMI						+0.32^**^				+0.47^**^			
			Longer-term (baseline vs. FU2) change in zBMI						+0.57^**^				+0.24^**^			
**Parental educational level**																
	**Lower edu**.		**Higher edu**.		**Lower edu**.		**Higher edu**.		**Lower edu**.		**Higher edu**.		**Lower edu**.		**Higher edu**.	
	***M***	***SE***	***M***	***SE***	***M***	***SE***	***M***	***SE***	***M***	***SE***	***M***	***SE***	***M***	***SE***	***M***	***SE***
Baseline	0.08	0.12	−0.22	0.11	−0.18	0.12	0.02	0.12	0.06	0.09	−0.21	0.09	−0.17	0.09	0.01	0.09
Follow-up 1	0.33	0.14	0.11	0.13	0.39	0.14	0.32	0.13	0.34	0.09	0.15	0.09	0.45	0.09	0.34	0.09
Follow-up 2	0.49	0.16	0.31	0.14	0.11	0.15	0.37	0.15	0.57	0.09	0.41	0.09	0.13	0.09	0.19	0.09
**Pairwise comparisons**			Shorter-term (baseline vs. FU1) change in zBMI						+0.28^**^		+0.36^**^		+0.62^**^		+0.32^**^	
			Longer-term (baseline vs. FU2) change in zBMI						+0.51^**^		+0.62^**^		+0.31^**^		+0.18^**^	
**Parental BMI**																
	**Lower BMI**		**Higher BMI**		**Lower BMI**		**Higher BMI**		**Lower BMI**		**Higher BMI**		**Lower BMI**		**Higher BMI**	
	***M***	***SE***	***M***	***SE***	***M***	***SE***	***M***	***SE***	***M***	***SE***	***M***	***SE***	***M***	***SE***	***M***	***SE***
Baseline	−0.11	0.11	−0.05	0.11	−0.05	0.12	−0.11	0.12	−0.10	0.09	−0.06	0.09	−0.04	0.09	−0.12	0.09
Follow-up 1	0.09	0.14	0.34	0.14	0.30	0.14	0.41	0.14	0.14	0.09	0.34	0.09	0.31	0.09	0.47	0.09
Follow-up 2	0.30	0.15	0.52	0.17	0.26	0.16	0.21	0.16	0.38	0.09	0.59	0.09	0.16	0.09	0.15	0.09
**Pairwise comparisons**			Shorter-term (baseline vs. FU1) change in zBMI						ns		ns		ns		ns	
			Longer-term (baseline vs. FU2) change in zBMI						+ 0.48^**^		+0.65^**^		+ 0.20^**^		+0.27^**^	
**Parental depressive symptoms**											
	**Lower depress**.		**Higher depress**.		**Lower depress**.		**Higher depress**.		**Lower depress**.		**Higher depress**.		**Lower depress**.		**Higher depress**.	
	***M***	***SE***	***M***	***SE***	***M***	***SE***	***M***	***SE***	***M***	***SE***	***M***	***SE***	***M***	***SE***	***M***	***SE***
Baseline	−0.13	0.12	−0.03	0.11	−0.02	0.12	−0.14	0.12	−0.15	0.09	−0.02	0.08	−0.02	0.09	−0.15	0.09
Follow-up 1	0.05	0.13	0.37	0.13	0.36	0.14	0.35	0.15	0.07	0.09	0.37	0.08	0.37	0.09	0.41	0.09
Follow-up 2	0.34	0.15	0.47	0.14	0.14	0.15	0.35	0.17	0.41	0.09	0.54	0.08	0.05	0.09	0.28	0.09
**Pairwise comparisons**			Shorter-term (baseline vs. FU1) change in zBMI						ns		ns		ns		ns	
			Longer-term (baseline vs. FU2) change in zBMI						+0.56^**^		+0.56^**^		+0.07^*^		+0.43^**^	

Significance levels: ^*^p < 0.01, ^**^p < 0.001. Pairwise comparisons are presented only for significant interaction effects. Comparisons for non-significant interactions are indicated by ns.

### 3.6. Sensitivity analyses

To assess the robustness of the findings, the four models were tested again twice using both the non-imputed and imputed data: first, without multivariate outliers; and second, including only parents in the app condition who completed at least 1 lesson or challenge. In the non-imputed dataset, 17 multivariate outliers (i.e., cases with a Mahalanobis distance >14; this cut-off was based on a chi-square distribution with df = 3 and *p* = 0.003) and 56 parents who did not complete any lesson or challenge were excluded for the sensitivity analyses. The pattern of statistically significant results of the models did not change compared to the primary analyses when the non-imputed data were analyzed without multivariate outliers and when only the parents who completed at least 1 lesson or challenge were included.

For the sensitivity analyses performed in the imputed data (i.e., containing 20 simulations of the original dataset), ~24 multivariate outliers per simulation (*M* = 24.35, *SD* = 3.50, range = 19–32) and a total of 56 parents who did not complete any lesson or challenge were excluded. In the analyses excluding multivariate outliers, the following two-way interactions were no longer significant: (1) the interaction between condition and the shorter-term time contrast in the model testing the overall intervention effect; (2) the interaction between condition and the shorter-term time contrast and the interaction between education and the shorter-term time contrast in the model testing the moderating effect of parental educational level; (3) the interaction between condition and the shorter-term time contrast in the model testing the moderating effect of parental BMI; and (4) the interaction between condition and the shorter-term time contrast in the model testing the moderating effect of parental depressive symptoms. The pattern of statistically significant three-way interactions (i.e., between condition, both time contrasts, and each of the moderators: parental educational level, BMI, and depressive symptoms) did not change compared to the primary analyses. Moreover, in the analyses including only parents who completed at least 1 lesson or challenge, no differences in the pattern of statistically significant effects were observed.

### 3.7. Exploratory analyses

To examine whether parents in the app condition (*n* = 177 in total) that were included in the adjusted PP analyses (*n* = 47) differed from parents who did not adhere to the criteria for higher app use (*n* = 130), a series of independent samples *t*-tests were performed in the non-imputed data. Parents who adhered to the criteria for higher app use were more likely to recommend the app to others (*M* = 6.45, *SD* = 1.97; on a scale from 1 to 10) than parents who did not adhere to these criteria (*M* = 5.14, *SD* = 2.31), *t*_(109)_ = −3.03, *p* = 0.003. Parents who adhered to the criteria for higher app use did not differ from parents who did not adhere to these criteria based on other app use characteristics (i.e., app rating, general app use, general skills in using apps, and subjective importance of apps), nor did they differ on several parent (i.e., age, educational level, financial difficulty, BMI, and depressive symptoms) and child (i.e., age, sex, being first-born, zBMI) characteristics (all *p*'s > 0.05).

Tables 6, 7 present the results of the adjusted PP analyses examining intervention effects in the non-imputed and imputed data. The results of the adjusted PP analyses were mostly in line with the findings of the main ITT analyses. With respect to the overall intervention effect, similar main effects of both time contrasts and two-way interaction effects between condition and both time contrasts were found, using both the non-imputed and imputed data (see Table 6). With respect to the subgroup analyses based on parental educational level, BMI, and depressive symptoms, two differences in effects (using the imputed data) were observed in the adjusted PP analyses. First, unlike in the ITT analyses, the three-way interaction between parental educational level, condition, and the longer-term time contrast was no longer statistically significant in the adjusted PP analyses. Second, we found a statistically significant three-way interaction between parental BMI, condition, and the shorter-term time contrast in the adjusted PP analyses, that had not emerged as statistically significant in the ITT analyses. The estimated means and standard errors in Table 7 show that when comparing parents in the control condition to those in the app condition with higher app use, the increases in zBMI were greatest among children of parents in the control condition who had higher BMI. All other three-way interaction effects that were statistically significant in the ITT analyses (i.e., between parental educational level, condition, and shorter-term time contrast; parental BMI, condition, and the longer-term time contrast; and parental depressive symptoms, condition, and the longer-term time contrast) were observed in the same direction in the adjusted PP analyses and showed identical patterns of mean-level differences (see Table 7).

**Table 6:** Adjusted PP intervention effects on child zBMI for subgroups based on parental educational level, BMI, and depressive symptoms in the non-imputed and imputed data.

	**Non-imputed**				**Imputed**			
	***b***	***SE***	**95% CI**	***p***	***b***	***SE***	**95% CI**	***p***
**Overall intervention effect**				
Condition	−0.31	0.17	−0.62 to 0.01	0.065	−0.21	0.13	−0.47 to 0.05	0.115
Shorter-term (baseline vs. FU1)	0.27	0.09	0.08–0.46	**0.004**	0.40	0.02	0.36–0.43	**< 0.001**
Longer-term (baseline vs. FU2)	0.37	0.10	0.19–0.58	**< 0.001**	0.41	0.02	0.37–0.43	**< 0.001**
Condition * shorter-term	−0.31	0.19	−0.66 to 0.10	0.103	−0.15	0.03	−0.22 to −0.08	**< 0.001**
Condition * longer-term	0.11	0.20	−0.26 to 0.50	0.580	0.34	0.03	0.27–0.40	**< 0.001**
**Parental educational level**				
Condition	−0.32	0.17	−0.65 to 0.03	0.053	−0.22	0.13	−0.48 to 0.04	0.095
Shorter-term (baseline vs. FU1)	0.27	0.10	0.09–0.44	**0.005**	0.40	0.02	0.37–0.43	**< 0.001**
Longer-term (baseline vs. FU2)	0.37	0.10	0.13–0.56	**< 0.001**	0.40	0.02	0.37–0.44	**< 0.001**
Parental educational level	−0.17	0.17	−0.02 to 0.03	0.551	−0.17	0.13	−0.43 to 0.08	0.188
Condition * shorter-term	−0.34	0.19	−0.68 to 0.04	0.092	−0.14	0.03	−0.22 to −0.08	**< 0.001**
Condition * longer-term	0.10	0.20	−0.35 to 0.52	0.730	0.33	0.03	0.26–0.40	**< 0.001**
Condition * parental educational level	−0.59	0.33	−0.04 to 0.07	0.556	−0.43	0.26	−0.94 to 0.09	0.104
Shorter-term * parental educational level	0.03	0.19	−0.03 to 0.04	0.737	−0.11	0.03	−0.18 to −0.04	**0.002**
Longer-term * parental educational level	−0.22	0.20	−0.02 to 0.05	0.395	−0.14	0.03	−0.21 to −0.07	**< 0.001**
Condition * shorter-term * Parental educational level	0.59	0.38	−0.09 to 0.05	0.602	0.35	0.07	0.22–0.49	**< 0.001**
Condition * longer-term * Parental educational level	−0.51	0.40	−0.04 to 0.08	0.394	−0.06	0.07	−0.19 to 0.08	0.422
**Parental BMI**				
Condition	−0.33	0.17	−0.19 to 0.23	0.051	−0.22	0.13	−0.48 to 0.04	0.100
Shorter-term (baseline vs. FU1)	0.27	0.10	0.24–0.47	**0.005**	0.40	0.02	0.36–0.43	**< 0.001**
Longer-term (baseline vs. FU2)	0.35	0.10	0.26–0.55	**< 0.001**	0.40	0.02	0.37–0.43	**< 0.001**
Parental BMI	0.01	0.01	−0.01 to 0.03	0.551	0.01	0.01	−0.01 to 0.03	0.471
Condition * shorter-term	−0.32	0.19	−0.41 to 0.11	0.254	−0.16	0.03	−0.23 to −0.09	**< 0.001**
Condition * longer-term	0.07	0.21	−0.12 to 0.41	0.234	0.32	0.03	0.25–0.39	**< 0.001**
Condition * parental BMI	0.02	0.03	−0.02 to 0.06	0.427	0.01	0.02	−0.03 to 0.05	0.619
Shorter-term * parental BMI	0.01	0.02	−0.01 to 0.04	0.189	0.01	0.00	0.01–0.02	**< 0.001**
Longer-term * parental BMI	0.01	0.02	−0.02 to 0.03	0.641	0.01	0.00	0.01–0.02	**< 0.001**
Condition * shorter-term * Parental BMI	−0.02	0.03	−0.04 to 0.06	0.934	−0.02	0.00	−0.03 to 0.004	**0.006**
Condition * longer-term * parental BMI	0.03	0.03	−0.04 to 0.07	0.619	0.01	0.00	0.0003–0.02	**0.045**
**Parental depressive symptoms**				
Condition	−0.32	0.17	−0.68 to 0.06	0.059	−0.21	0.13	−0.47–0.05	0.113
Shorter-term (baseline vs. FU1)	0.27	0.10	0.08–0.47	**0.004**	0.40	0.02	0.36–0.43	**< 0.001**
Longer-term (baseline vs. FU2)	0.37	0.10	0.15–0.58	**< 0.001**	0.41	0.02	0.37–0.44	**< 0.001**
Parental depressive symptoms	0.00	0.02	−0.04 to 0.05	0.953	0.01	0.02	−0.02 to 0.04	0.611
Condition * shorter-term	−0.32	0.19	−0.72 to 0.09	0.093	−0.15	0.03	−0.22 to −0.09	**< 0.001**
Condition * longer-term	0.09	0.21	−0.32 to 0.49	0.650	0.33	0.03	0.27–0.40	**< 0.001**
Condition * parental depressive symptoms	−0.00	0.05	−0.09 to 0.08	0.961	0.01	0.03	−0.06 to 0.07	0.841
Shorter-term * parental depressive symptoms	0.02	0.03	−0.04 to 0.07	0.525	0.02	0.00	0.01–0.03	**< 0.001**
Longer-term * parental depressive symptoms	0.01	0.03	−0.05 to 0.06	0.870	0.03	0.00	0.02–0.03	**< 0.001**
Condition * shorter-term * parental depressive symptoms	0.01	0.06	−0.09 to 0.12	0.806	−0.01	0.00	−0.02 to 0.01	0.506
Condition * longer-term * parental depressive symptoms	−0.06	0.06	−0.18 to 0.04	0.272	−0.03	0.00	−0.05 to −0.02	**< 0.001**

b, unstandardized regression coefficient; SE, standard error; CI, confidence interval. Parameter values for b and SE < 0.01 are presented as 0.00. Statistically significant effects (*p* < 0.05) are bolded.

**Table 7:** Estimated means (adjusted PP) for child zBMI at baseline, follow-up 1, and follow-up 2 for the app and control condition, specified by the levels (i.e., lower vs. higher) of parental educational level, BMI, and depressive symptoms in the non-imputed and imputed data.

	**Non-imputed**								**Imputed**							
	**App condition**				**Control condition**				**App condition**				**Control condition**			
**Overall intervention effect**																
	***M***		***SE***		***M***		***SE***		***M***		***SE***		***M***		***SE***	
Baseline	−0.32		0.16		−0.08		0.08		−0.35		0.12		−0.08		0.06	
Follow-up 1	−0.20		0.19		0.35		0.09		−0.03		0.12		0.39		0.06	
Follow-up 2	0.10		0.20		0.23		0.10		0.23		0.12		0.16		0.06	
**Pairwise comparisons**		Shorter-term (baseline vs. FU1) change in zBMI						+0.32^**^				+0.47^**^			
		Longer-term (baseline vs. FU2) change in zBMI						+0.58^**^				+0.24^**^			
**Parental educational level**																
	**Lower edu**.		**Higher edu**.		**Lower edu**.		**Higher edu**.		**Lower edu**.		**Higher edu**.		**Lower edu**.		**Higher edu**.	
	***M***	***SE***	***M***	***SE***	***M***	***SE***	***M***	***SE***	***M***	***SE***	***M***	***SE***	***M***	***SE***	***M***	***SE***
Baseline	−0.12	0.22	−0.54	0.22	−0.18	0.12	0.02	0.11	−0.18	0.16	−0.54	0.17	−0.17	0.09	0.01	0.09
Follow-up 1	−0.18	0.29	−0.28	0.25	0.39	0.13	0.32	0.13	0.11	0.16	−0.18	0.17	0.45	0.09	0.34	0.09
Follow-up 2	0.54	0.28	−0.35	0.28	0.12	0.14	0.35	0.14	0.47	0.16	−0.05	0.17	0.13	0.09	0.19	0.09
**Pairwise comparisons**		Shorter-term (baseline vs. FU1) change in zBMI						+0.28^**^		+0.36^**^		+0.62^**^		+0.32^**^	
		Longer-term (baseline vs. FU2) change in zBMI						ns		ns		ns		ns	
**Parental BMI**																
	**Lower BMI**		**Higher BMI**		**Lower BMI**		**Higher BMI**		**Lower BMI**		**Higher BMI**		**Lower BMI**		**Higher BMI**	
	***M***	***SE***	***M***	***SE***	***M***	***SE***	***M***	***SE***	***M***	***SE***	***M***	***SE***	***M***	***SE***	***M***	***SE***
Baseline	−0.37	0.21	−0.28	0.19	−0.05	0.12	−0.10	0.12	−0.38	0.16	−0.33	0.14	−0.04	0.09	−0.12	0.09
Follow-up 1	−0.24	0.26	−0.19	0.26	0.30	0.14	0.41	0.13	−0.09	0.16	0.03	0.14	0.31	0.09	0.47	0.09
Follow-up 2	−0.14	0.28	0.24	0.24	0.26	0.16	0.21	0.15	0.09	0.16	0.33	0.14	0.16	0.09	0.15	0.09
**Pairwise comparisons**			Shorter-term (baseline vs. FU1) change in zBMI						+0.28^**^		+0.30^**^		+0.27^**^		+0.59^**^	
			Longer-term (baseline vs. FU2) change in zBMI						+0.47^**^		+0.66^**^		+0.20^**^		+0.27^**^	
**Parental depressive symptoms**				
	**Lower depress**.		**Higher depress**.		**Lower depress**.		**Higher depress**.		**Lower depress**.		**Higher depress**.		**Lower depress**.		**Higher depress**.	
	***M***	***SE***	***M***	***SE***	***M***	***SE***	***M***	***SE***	***M***	***SE***	***M***	***SE***	***M***	***SE***	***M***	***SE***
Baseline	−0.33	0.24	−0.32	0.23	−0.02	0.11	−0.14	0.12	−0.37	0.18	−0.33	0.17	−0.02	0.09	−0.15	0.09
Follow-up 1	−0.32	0.31	−0.10	0.31	0.37	0.13	0.35	0.14	0.11	0.18	0.05	0.17	0.37	0.09	0.41	0.09
Follow-up 2	0.21	0.31	−0.01	0.32	0.15	0.15	0.35	0.16	0.16	0.18	0.27	0.17	0.05	0.09	0.28	0.09
**Pairwise comparisons**			Shorter-term (baseline vs. FU1) change in zBMI						ns		ns		ns		ns	
			Longer-term (baseline vs. FU2) change in zBMI						+0.53^**^		+0.60^**^		+0.07^*^		+0.43^**^	

Significance levels: ^*^p < 0.01, ^**^p < 0.001. Pairwise comparisons are presented only for significant interaction effects. Comparisons for non-significant interactions are indicated by ns.

## 4. Discussion

This study investigated the process and effectiveness of the app-based parenting program *Samen Happie!* on child zBMI at 6 and 12-months follow-up. Process data showed that app acceptability was moderate, but that sustained app use was low. ITT analyses with imputed data revealed that zBMI increased in both conditions, but that this increase in zBMI was least pronounced in the app condition at the 6-month follow-up, particularly among children of parents with lower educational level. These effects were further supported by exploratory PP analyses focusing on parents with higher app use. In addition, adjusted PP analyses suggested beneficial shorter-term effects of higher app use for children of parents with higher BMI when compared with children of parents with higher BMI in the control condition. Despite these positive effects at the shorter-term follow-up, greater increases in zBMI were observed in the app condition at the 12-month follow-up in general. Overall, our findings suggest that the *Samen Happie!* app might prevent increases in zBMI of young children in the shorter-term, particularly among children of parents with lower educational level and parents with higher BMI who used the app more frequently, but that (even higher) app use does not appear to prevent increases in zBMI on the longer-term.

Across conditions, we found a general trend of increased zBMI over time in our sample of Dutch 0-to-2-year-olds, with increases from −0.09 at 10 months of age to 0.31 at 15.5 months, and to 0.39 at 23 months. Similar zBMI trajectories in this age group were observed in a Dutch (61) and a Canadian (62) study that used the same reference population (63). As such, patterns of increasing zBMI in this age group seem to be a common characteristic unspecific to our study.

The shorter-term finding that particularly children of parents with lower educational level and higher BMI (who used the app more frequently) profited most from the *Samen Happie!* program after 6 months, was in line with our hypotheses. However, these effects seemed to diminish at the longer-term, with the overall app condition showing higher zBMI values after 12 months compared to the control condition. Particularly shorter-term effects have been found before in digital preventive interventions for obesity [including interventions targeting children/adolescents and parents; (64)] and early interventions for high-risk infants (65). When looking specifically at mHealth parenting programs (i.e., using mobile systems such as apps, websites, and text messaging) for the prevention or treatment of childhood obesity, a recent review found mostly no effects on child zBMI (also not at the shorter-term), however, these studies mainly included older children (66). One app-based program among preschoolers (MINISTOP) that was included in this review showed effects on a composite score including fat mass index and dietary and physical activity variables after 6 months [but not fat mass only at 6 months; (67)], but these effects were not retained at the 12-month follow-up (68). We identified only one other app-based parenting program for obesity prevention [Growing Healthy; not included in review (66)] that examined weight outcomes in infants, finding no effects on child zBMI, however, children in this study were somewhat younger at follow-up (69). Overall, our findings are in line with these previous findings suggesting that the effects of mHealth parenting programs on child weight status to date may be limited and fading over time.

We do not have an explanation for these longer-term fading effects, other than speculating about potential rebound effects when app use decreased over time. Of note, decreased app use probably forms the most important explanation for the finding that children of parents with lower educational level and higher BMI (that use the app more frequently) seem to have shortly profited from the app, but not on the longer-term. Importantly, process data indicated that most app use—even among parents who used the app more frequently—occurred at the shorter-term (i.e., between baseline and the 6-month follow-up), which supports the notion that active, sustained use of the app is probably needed for longer-term effects to establish. Additionally, our process data indicated that parents completed relatively more lessons than challenges, and that even among the more frequent app users, only two-thirds of parents completed at least half of the challenges. Whereas, the lessons focused primarily on enhancing knowledge and attitudes through behavior changes techniques like consciousness raising and framing, the challenges were designed to facilitate the transfer of this knowledge into regular daily habits [e.g., through implementation intentions; see (40)]. Hence, longer-term effects may depend on parents using the app, and particularly the challenges, more intensely.

Our findings suggest that engaging frequently with an app is important for the effectiveness of app-based programs [see also the review by Rossiter et al. (65)]. Although we deliberately gave no instructions regarding the timing and frequency of app use with the goal to mimic actual program adherence in real life, this might have resulted in the observed patterns of declining and generally low app use. A pattern of decreasing app use within the first weeks is frequently observed in app-based health interventions (70) and might be caused by a drop in engagement when the novelty of the app wears off (71). The moderate levels of app acceptability in combination with parents reporting to use the app mainly after installing it (but not frequently anymore after that), indicate that our results are in line with these previous findings. A recent review among a broad range of app-based health programs showed that the programs with the highest user engagement were primarily characterized by the option to receive (particularly personalized) push notifications, easy access to information, and the ability to communicate with health professionals (70). Although our participants were able to receive personalized push notifications for the challenges, this feature could have been made stronger if we had used it regularly (e.g., weekly) as a reminder for lessons and challenges that parents had not yet completed. Moreover, access to the information in our app was relatively easy through the age- and theme-based content, but our process data suggested that some parents would prefer a solely theme-based structure organized around child EBRBs (e.g., dietary intake, sleep) over the overarching age-based modules. From an engagement perspective, a potential downside of the age-based modules might have been that parents could not explore content for the next developmental stages of their child until their child reached the minimum age of that level. This might have impeded the eagerness of interested and motivated parents as well as potential positive anticipatory guidance [i.e., proactive advice effects (72, 73)]. Lastly, our app offered parents digital parenting support without the option for direct communication with health professionals, whereas additional support in the form of (offline) health-professional led support groups might have increased engagement (74, 75). Strategies combining easily accessible parenting apps with additional (offline) support might be particularly helpful to stimulate longer-term benefits of the *Samen Happie!* app among at-risk families that need more tailored parenting support. Future research should corroborate whether these strategies match parents' preferences for app-based parenting programs and whether they can stimulate parental engagement over longer periods of time.

### 4.1. Limitations and directions for future research

Several strengths of this study should be noted, including that it was pre-registered, conducted in a representative sample of Dutch adults (aged 25– 45 years) in terms of educational level (76), and had high retention rates (almost 90% at 12-month follow-up). Nevertheless, the study also has limitations. First, although the patterns of results in the imputed and non-imputed data were largely similar, we only found statistically significant effects in the imputed data. Even though the quality of the imputed values was good and most findings were corroborated in the exploratory PP analyses, the results need to be interpreted with caution and confirmed by future studies. That findings were not statistically significant when using the non-imputed data might be due to the loss of statistical power caused by the high number of missing values in child zBMI (64% at 12-month follow-up). To increase the validity of the data, we asked parents to report child height and weight as measured at the preventive child health clinic, but this resulted in a great deal of missingness because children were only measured at specific time points. Missingness in child zBMI did not depend on characteristics other than child age and the chances of bias are therefore low, but the high number of missingness could have posed power issues to detect effects. Future studies should line up the assessment of child height and weight with visits to the preventive child health clinic to ensure complete anthropometric outcome data.

Second, we examined child zBMI as primary and sole outcome. Although zBMI is a sex- and age-specific measure, the wide age range at baseline (5–15 months) might have potentially influenced our results, but we were unable to test this due to power restrictions. The use of zBMI to measure weight status in infancy is recommended by pediatric societies (77, 78) and this measure shows consistent links with adiposity in childhood (62), however, some have argued that BMI alone does not provide the best indication of adiposity [e.g., (79)]. Particularly in the first years of life, rapid changes in body composition (e.g., fluid balance) can result in changes in fat (free) mass, and these processes can vary greatly between children (80). Moreover, intervention-induced changes in healthy EBRBs might not always be visible through changes in BMI (79), particularly in interventions shorter than 12 months (81). Together with the complexity of infant weight development, this emphasizes the need to assess other outcomes related to children's energy balance, such as dietary intake, sleep, and physical (in)activity in addition to zBMI.

Third, although parental educational level is a frequently used indicator of family SEP in pediatric health research (82), it was the only indicator of SEP we used in this study. There might be other relevant socioeconomic factors that also play a role in child health outcomes, such as family income, parental employment, and the neighborhood a family lives (83, 84). As associations between indicators of SEP can be low (85, 86), each of those indicators individually as well as the interaction between indicators could importantly influence a family's SEP and is therefore interesting to investigate in future parenting research.

Fourth, although our sample was representative in terms of parental educational level, the sample was homogeneous in terms of ethnicity, with more than 95% of parents being born in the Netherlands. However, no other indicators of cultural background were assessed such as religion or language(s) spoken at home, whereas such factors might have affected program engagement and effectiveness (87). Future obesity prevention programs should recruit a diverse population of participants in terms of ethnic and cultural background. Additionally, programs should consider recruiting first-time parents in particular given that these parents might have a higher need for parenting support, as indicated by the higher levels of engagement of first-time parents in a healthy feeding intervention (88).

Fifth and finally, only primary caregivers participated in this study, which were primarily mothers (>95%). However, other caregivers might also be involved in energy balance-related parenting, such as fathers (89) and grandparents [who might even promote unhealthy dietary intake and weight status; (90)]. As one third of infants in a Dutch study was cared for by others (e.g., grandparents, daycare) for more than 20 h per week (61), it is important that future preventive interventions for childhood obesity also target other caregivers that are involved in energy balance-related parenting.

## Conclusion

In conclusion, this study showed that the app-based parenting program *Samen Happie!* might be effective in preventing increases in infant zBMI after 6 months, particularly among children of parents with lower educational level and children of parents with higher BMI who use the app more frequently. Despite these promising effects at the shorter-term, however, greater increases in zBMI were observed among children of parents who used the app after 12 months. Future research should be directed at replicating the positive effects found after 6 months and at finding ways to extend these effects to the longer-term. To this end, it is imperative to determine what is needed to stimulate sustained app use and engagement in mHealth parenting programs for childhood obesity and how these programs can be complemented with additional (offline) support for high-risk families in particular.

## Data availability statement

The datasets presented in this study can be found in online repositories. The names of the repository/repositories and accession number(s) can be found below: https://osf.io/wcsxg/ (Open Science Framework).

## Ethics statement

The studies involving human participants were reviewed and approved by the Ethics Committee of the Faculty of Social Sciences, Radboud University, the Netherlands. Written informed consent to participate in this study was provided by the participants' legal guardian/next of kin.

## Author contributions

JL, LK, JV, CW, RH, ER, and SK contributed to the conceptualization of the study and development of the *Samen Happie!* program. LK, JL, JV, and CW developed the questionnaires. LK managed data collection. LK with input from WB, performed data analyses. LK drafted the paper, on which all authors provided critical feedback. All authors contributed to the article and approved the submitted version.
