# Supplementary material for: Process and effect evaluation of the app-based parenting program Samen Happie! on infant zBMI: A randomized controlled trial
Source: Front Public Health. 2022 Dec 23;10:1012431. doi: 10.3389/fpubh.2022.1012431 (PMC9822729; doi:10.3389/fpubh.2022.1012431)
Supplement: Supplementary file 1 [file Table_1.DOCX]

Supplemental Table 1: Number of Available Lessons and Challenges and Mean Number of Completed Lessons and Challenges per Theme and Per Module Derived from the App Use Database

| Module | 1 | 2 | 3 | 4 | 5 |  |
| --- | --- | --- | --- | --- | --- | --- |
| Child age (in months) | 7-12 | 12-15 | 15-18 | 18-24 | 24-48 |  |
| **Number of lessons and challenges per theme (*N* = 123^a^)** | | | | | | **Total** |
| Food | 5 | 3 | 3 | 4 | 5 | 20 |
| (lessons/challenges) | (4/1) | (2/1) | (2/1) | (3/1) | (4/1) | (15/5) |
| *M* (*SD*) lessons | 2.60 (1.58) | 0.81 (0.95) | 0.50 (0.85) | 0.39 (0.98) | 0.05 (0.34) | 4.35 (3.09) |
| *M* (*SD*) challenges | 0.58 (0.50) | 0.36 (0.48) | 0.22 (0.41) | 0.11 (0.31) | 0.01 (0.09) | 1.27 (1.21) |
| Drinks | 4 | 2 | 2 | 2 | 4 | 14 |
| (lessons/challenges) | (3/1) | (1/1) | (2/0) | (2/0) | (4/0) | (12/2) |
| *M* (*SD*) lessons | 1.47 (1.37) | 0.33 (0.47) | 0.43 (0.81) | 0.25 (0.66) | 0.00 (0.00) | 2.49 (2.43) |
| *M* (*SD*) challenges | 0.55 (0.50) | 0.31 (0.46) | - | - | - | 0.86 (0.77) |
| Sleep | 3 | 0 | 3 | 1 | 3 | 10 |
| (lessons/challenges) | (2/1) | - | (2/1) | (1/0) | (2/1) | (7/3) |
| *M* (*SD*) lessons | 0.99 (0.94) | - | 0.42 (0.79) | 0.12 (0.33) | 0.00 (0.00) | 1.54 (1.53) |
| *M* (*SD*) challenges | 0.42 (0.50) | - | 0.18 (0.38) | - | 0.00 (0.00) | 0.60 (0.71) |
| Screen time/PA | 0 | 3 | 0 | 3 | 2 | 8 |
| (lessons/challenges) | - | (2/1) | - | (2/1) | (2/0) | (6/2) |
| *M* (*SD*) lessons | - | 0.55 (0.86) | - | 0.20 (0.58) | 0.02 (0.18) | 0.76 (1.18) |
| *M* (*SD*) challenges | - | 0.27 (0.44) | - | 0.10 (0.30) | - | 0.37 (0.60) |
| Summary (lessons only) | 1 | 1 | 1 | 0 | 0 | 3 |
| *M* (*SD*) lessons | 0.37 (0.49) | 0.24 (0.43) | 0.17 (0.38) | - | - | 0.78 (0.99) |
| Temper (lessons only) | 0 | 0 | 0 | 1 | 2 | 3 |
| *M* (*SD*) lessons | - | - | - | 0.12 (0.33) | 0.00 (0.00) | 0.12 (0.33) |
| Parental wellbeing (lessons only) | 2 | 2 | 2 | 3 | 3 | 12 |
| *M* (*SD*) lessons | 0.93 (0.96) | 0.46 (0.84) | 0.44 (0.81) | 0.28 (0.86) | 0.02 (0.20) | 2.14 (2.49) |
| **Number of lessons and challenges per module (*N* = 123^a^)** | | | | | | **Total** |
| Lessons | 12 | 8 | 9 | 12 | 17 | 58 |
| *M* (*SD*) completed | 6.37 (4.67) | 2.40 (3.17) | 1.96 (3.45) | 1.36 (3.57) | 0.09 (0.67) | 12.18 (11.12) |
| Challenges | 3 | 3 | 2 | 2 | 2 | 12 |
| *M* (*SD*) completed | 1.55 (1.29) | 0.93 (1.29) | 0.40 (0.78) | 0.20 (0.57) | 0.01 (0.09) | 3.10 (2.89) |
| Total | 15 | 11 | 11 | 14 | 19 | 70 |
| Cumulative | 15 | 26 | 37 | 51 | 70 | 70 |
| **Number of parents in highest available module** | | | | | | **Total** |
| At T0 | 135 | 40 | 2 | 0 | 0 | 177 |
| Percentage of total | 76.3% | 22.6% | 1.1% | 0.0% | 0.0% | 100% |
| At T1 | 5 | 75 | 39 | 38 | 0 | 157 |
| Percentage of total | 3.2% | 47.8% | 24.8% | 24.2% | 0.0% | 100% |
| At T2 | 0 | 0 | 0 | 94 | 56 | 150 |
| Percentage of total | 0.0% | 0.0% | 0.0% | 62.7% | 37.3% | 100% |

Note. ^a^ Total unique users of the app.
